# A Psychometric Analysis of the Italian Version of the eHealth Literacy Scale Using Item Response and Classical Test Theory Methods

**DOI:** 10.2196/jmir.6749

**Published:** 2017-04-11

**Authors:** Nicola Diviani, Alexandra Lelia Dima, Peter Johannes Schulz

**Affiliations:** ^1^ Department of Health Sciences & Health Policy Faculty of Humanities and Social Sciences University of Lucerne Lucerne Switzerland; ^2^ Person Centered Health Care & Health Communication Group Human Functioning Unit Swiss Paraplegic Research Nottwil Switzerland; ^3^ Amsterdam School of Communication Research Faculty of Social and Behavioural Sciences University of Amsterdam Amsterdam Netherlands; ^4^ Health Services and Performance Research (HESPER EA 7425) University Claude Bernard Lyon 1 Lyon France; ^5^ Institute of Communication and Health Faculty of Communication Sciences Università della Svizzera italiana Lugano Switzerland

**Keywords:** eHealth literacy, eHEALS, item response theory, classical test theory, validation, Italian

## Abstract

**Background:**

The eHealth Literacy Scale (eHEALS) is a tool to assess consumers’ comfort and skills in using information technologies for health. Although evidence exists of reliability and construct validity of the scale, less agreement exists on structural validity.

**Objective:**

The aim of this study was to validate the Italian version of the eHealth Literacy Scale (I-eHEALS) in a community sample with a focus on its structural validity, by applying psychometric techniques that account for item difficulty.

**Methods:**

Two Web-based surveys were conducted among a total of 296 people living in the Italian-speaking region of Switzerland (Ticino). After examining the latent variables underlying the observed variables of the Italian scale via principal component analysis (PCA), fit indices for two alternative models were calculated using confirmatory factor analysis (CFA). The scale structure was examined via parametric and nonparametric item response theory (IRT) analyses accounting for differences between items regarding the proportion of answers indicating high ability. Convergent validity was assessed by correlations with theoretically related constructs.

**Results:**

CFA showed a suboptimal model fit for both models. IRT analyses confirmed all items measure a single dimension as intended. Reliability and construct validity of the final scale were also confirmed. The contrasting results of factor analysis (FA) and IRT analyses highlight the importance of considering differences in item difficulty when examining health literacy scales.

**Conclusions:**

The findings support the reliability and validity of the translated scale and its use for assessing Italian-speaking consumers’ eHealth literacy.

## Introduction

### Health Information on the Web

Following the advent of the Internet, health-related information is increasingly available to the public [1]. It has been estimated that almost 3 out of 4 Internet users worldwide have looked for health information on the Web [2,3]. Wrong or incomplete information could potentially have negative consequences, for instance, on the doctor-patient relationship, participation in screening programs, or adherence to treatments [4]. More attention needs therefore to be devoted to people’s ability to interact with Web-based health information.

People’s general ability to deal with health information has traditionally been defined as health literacy [5]. To assess health literacy skills in the electronic environment, Norman and Skinner [6,7] have introduced the concept and the measure of eHealth literacy, defined as “the ability to seek, find, understand, and appraise health information from electronic sources and apply the knowledge gained to addressing or solving a health problem.” According to its authors, the eHealth Literacy Scale (eHEALS) is a promising tool to assess consumers’ comfort and skills in using information technology for health and to identify those who may benefit from referrals to eHealth interventions or resources within a clinical environment [7].

### Translations of the eHealth Literacy Scale

eHEALS consists of 8 items measuring consumers’ combined knowledge, comfort, and perceived skills related to finding, evaluating, and applying electronic health information to health problems. The scale was developed building on the concept of eHealth literacy [7]. According to its authors, eHealth literacy comprises six core skills, or literacies (traditional literacy, health literacy, information literacy, scientific literacy, media literacy, and computer literacy) which, following principles of the social cognitive theory and self-efficacy theory [8], are to be considered precursors of behavior change and skill development [6,7].

The authors of the scale have demonstrated the reliability and validity of its original English version [7]. Over the years, other studies have supported the reliability and validity of eHEALS, for instance, by showing that the scale correlates strongly with—but is distinct from—several scales measuring different aspects of health-related Internet use, such as health information seeking on the Web, attitudes toward the adoption of information and communication technologies (ICTs) for health purposes, use of Internet searching strategies, perceived outcomes of seeking health information by surfing the net, and use of Internet evaluation criteria [7,9,10]. Across these studies, no consistent association of eHEALS scores with the personal characteristics of the respondents, such as gender, education, or age was found. eHEALS has so far been translated and validated in Dutch [11], Japanese [12], Chinese [13], German [14], Spanish [15], Italian [16], Iranian [17], and Hebrew [10,18]. All the linguistic versions of the scale presented high internal consistency measured via Cronbach alpha. These results have generally been taken as an indication of the reliability of the scale in the different cultural contexts.

### First Open Question: Population Validity

Most of the studies aimed at validating linguistic versions of eHEALS present at least two important limitations. First of all, whereas the English version of the scale has been applied in a variety of samples, validations have mostly been conducted among specific populations, for instance, students [13-16], young adults [17], patients [11], or seniors [19]. These samples only partly reflect the target population of the tool, that is, consumers of Web-based health information. To date, therefore, it is still not possible to draw general conclusions on the reliability and validity of eHEALS in broader samples.

### Second Open Question: Item Difficulty

A second important limitation of past validation studies resides in the widespread reliance on classical test theory (CTT) and factor analysis (FA) only. Like traditional health literacy and other ability tests, the items of eHEALS refer to skills of varying difficulty that may belong to a single eHealth literacy continuum (see Multimedia Appendix 1 for an overview of the items). For instance, whereas knowing how to find health-related information using the Internet (item 1) could be considered a basic skill, being able to distinguish good and bad health information found on the Internet (item 7) could be considered a more advanced skill; as such, a respondent’s agreement with item 7 may indicate more intense confidence in their own eHealth literacy than their agreement with item 1. Differences in item difficulty lead to different probabilities of their different response options being endorsed by respondents. This might be the reason behind the different conclusions on the factorial structure of the scale drawn by different authors over the years. Soellner and colleagues [20], for instance, used confirmatory factor analysis (CFA) to compare the 1-factor model based on Norman and Skinners’ [7] analyses with a 2-factor model specified a priori based on the content and wording of the items of the scale and on own previous research [14]. The results of their analyses indicated a better fit for the 2-factor model, supporting the division into two subscales: *Information Seeking* (items 1-5 and 8) and *Information Appraisal* (items 6 and 7). CFA was also applied by Neter et al [18] to the Hebrew translation of the scale. Their analyses confirmed the better fit of a 2-factor solution, but two factors were found to include different items (Factor 1: items 1, 2, and 4; Factor 2: items 3 and 5-8). More recently, the exploratory factor analysis (EFA) using principal components analysis (PCA) of an Iranian translation of the scale also suggested a 2-factor structure which groups items 1 and 2 in the first factor and all the other items in a second factor [17]. All the other translations of eHEALS confirmed the 1-factor structure proposed by the authors of the scale, although van der Vaart and colleagues [11] reported in their EFA using PCA, a second component with an eigenvalue of 1.1, which could support the existence of a second dimension.

Psychometrics literature acknowledges that, if items vary in difficulty, PCA might produce a multidimensional solution that groups together items of similar difficulties; although CFA is considered an informative test of structural validity if item properties are known and acceptable, item response theory (IRT) methods are recommended for examining dimensionality in this context [21,22]. A recent psychometric analysis of the original version of eHEALS used PCA to test unidimensionality although it presented a rather comprehensive parametric IRT exploration of item properties [23]. Yet, scale dimensionality can be appropriately tested within the IRT framework together with several other item properties. Two distinct approaches are available and can be compared for a better understanding of the concept. Nonparametric item response theory (NIRT) (ie, Mokken scale analysis, MSA) allows testing fit to a measurement model arguably most appropriate for eHealth literacy. The concept refers to relative differences between individuals; that is, a person who knows both to find information and to assess its quality is described as having higher literacy than a person who can find information without being able to assess it, but the difference between these two persons is not quantitative in nature. For such concepts, MSA would be a first choice, as it allows to investigate whether an item set measures ordinal differences between respondents regarding a latent trait (ie, ordinal measurement) [23,24]. By comparison, parametric IRT methods aim at a precise quantification of differences. For ordinal items as in eHEALS, the rating scale model (RSM) represents a more stringent set of requirements which, if met at item and respondent level, would represent proof of optimal measurement quality in terms of precision and parsimony, also described as “fundamental measurement” [25]. By testing both IRT models on the Italian version of the eHealth Literacy Scale (I-eHEALS), we can understand in more detail its psychometric properties, what inferences it can support, and what avenues of further development can be pursued for this operationalization of eHealth literacy.

### Aims of the Study

As an attempt to get new insights on the two main open questions on eHEALS outlined above, this paper reports on the translation and validation in a population sample of the I-eHEALS. In addition to CTT and FA, we applied MSA to examine dimensionality and model fit, and employed parametric IRT methods to reproduce and extend prior explorations of eHEALS item properties [23,26].

## Methods

### Overview

In order to explore the psychometric properties of I-eHEALS, a Web-based survey was conducted among a sample of individuals living in the Italian-speaking region of Switzerland. This population is very close to the Italian population from a sociocultural point of view and also as regards health information seeking activities on the Web, as they have access to the same information.

### Procedure and Participants

Data were collected through Web-based self-administered questionnaires in two surveys conducted within a larger project in Summer 2013 (Study 1) and Spring 2015 (Study 2). Participants for both surveys were recruited through advertisements placed in the waiting room of a medical private practice, at a local university, as well as in a regional Web-based newspaper. The choice of using different channels for recruitment had the objective to ensure diversity within the samples regarding age and educational background. The advertisements contained information about the study, contact details of the research team, and a link to the questionnaire. Participants could take part in the survey only once. All participants who completed the survey and agreed to provide contact details (email address or phone number) were entered into a prize draw to win one of three €25 coupons from a local grocery store. A total of 296 individuals (N_Study1_=117, N_Study2_=179) aged between 16 and 71 years (mean age 37.37, SD 13.776) comprised the final sample. The sample was predominantly female (193/296, 65.2%), and almost half of the respondents had at least some university education (129/296, 43.6%). The remaining respondents had either a high school diploma (82/296, 27.7%) or a vocational training certificate (55/296, 18.6%).

Whereas almost 9 out of 10 respondents (257/296, 86.8%) reported using the Internet every day, the majority of them (210/296, 70.9%) reported using it for health-related information less than once a week. No significant differences in Web-based health information seeking were observed between Study 1 and Study 2 (*P*=.44).

Participants to Study 1 (mean 33.81, SD 10.466) were slightly younger compared with those in Study 2 (mean 39.77, SD 15.170); *t*_288_=−3.697, *P*<.001, *d*=.46; however, the two samples did not differ as regards gender (χ^2^=4.5 *P*=.104) and educational level (χ^2^=.7, *P*=.88).

According to the Swiss Federal Act on Research involving Human Beings (Human Research Act [HRA], September 30, 2011), research not concerning diseases or the structure or the function of the human body does not need formal approval from an ethical review board. All participants were informed about the nature and the aims of the study before enrollment and could decide to withdraw their consent to study participation at any time.

### Instrument and Measures

The main section of the surveys was devoted to the 8 items of the I-eHealth Literacy Scale (Multimedia Appendix 1). Like in the English version of the scale, participants were asked to rate their agreement with the statements on a 5-point Likert scale ranging from *Strongly disagree* to *Strongly agree* [7]. The scale underwent a rigorous forward and backward translation process conducted in accordance with the World Health Organization guidelines [27]. In a first step, an Italian-speaking translator fluent in English and knowledgeable of the English-speaking culture translated the items into Italian. In a second step, the items were translated back to English by an independent translator, whose mother tongue was English and who had no knowledge of the questionnaire. The resulting items were compared with the original items by the two translators and the research team to identify possible conceptual differences. Additionally, in order to fully take into account possible cultural differences, in-depth interviews were conducted among 13 individuals considered representative of the target population. The sample of the in-depth interview was composed of 4 men and 9 women aged between 17 and 61 years, with varying levels of education and with different Internet usage habits. All participants to the interviews were instructed to think aloud during the completion of the questionnaire and to highlight problematic points. The whole process led to some minor changes in wording and confirmed the clarity and comprehensibility of I-eHEALS.

Only in Study 1, data were also collected about the respondents’ experiences with and attitudes toward health information seeking on the Web. In particular, data were collected about frequency of Web-based health information seeking, trust in the Internet as a source of health information, attitudes toward the adoption of ICTs for health purposes (2 items, *r*=.692, *P*<.001), use of Internet searching strategies (5 items, Cronbach alpha=.674) [10], perceived outcomes of seeking health information by surfing the net (9 items, Cronbach alpha=.937) [10,28], use of Internet evaluation criteria (5 items, Cronbach alpha=.879) [29], and predisposition toward eHealth in general (2 items, *r*=.600, *P*<.001) [7]. All these constructs are known to be positively related to eHealth literacy and were used to assess the convergent validity of the Italian scale. An overview of the scales used in the study is presented in Multimedia Appendix 2.

Finally, data about selected sociodemographic characteristics of the participants were collected. These included gender, age, educational level, and general and health-related Internet use.

### Data Analysis

Statistical analyses were conducted using IBM SPSS Statistics 21.0, R statistical software (R Foundation for Statistical Computing), and Winsteps software (Winsteps, Beaverton, Oregon).

#### Item Characteristics and Exploratory Factor Analysis

First, item characteristics were described. Factorability of the 8 I-eHEALS items was examined by computing inter-item correlations (all the items should correlate at least .3 with at least one other item), the Kaiser-Meyer-Olkin measure of sampling adequacy (recommended value >.6), and Bartlett’s test of sphericity (should be significant) [30]. PCA was subsequently conducted to examine the latent variables underlying the observed variables of the Italian scale [31] following previously used methods [11].

#### Confirmatory Factor Analysis

In a second step, in order to compare the unidimensional solution (Model I) proposed by Norman and Skinner [7] with the 2-factor solution identified by Soellner et al [14] and emerged during PCA of our own data (Model II), model fit indices for the two models were calculated using CFA. Model fit was tested using chi-square tests [32], and the following model fit indices and cutoff values: comparative fit index (CFI) >0.95, Tucker-Lewis index (TLI) >0.95, root mean squared error of approximation (RMSEA) <0.06, and standardized root mean squared residual (SRMR) <0.09 [33]. The two (nested) models were compared using the chi-square difference test (*Anova* function; *Lavaan* package [34]).

#### Item Response Theory Analyses

Subsequently, NIRT analyses (MSA) were performed using the *Mokken* package in R [35] to examine the structure of the scale taking into account variations in item difficulty (ie, differences between items in the proportion of answers indicating high ability). MSA examines whether an item set orders respondents accurately on a continuum representing a latent trait by testing unidimensionality (whether items can be located on a single latent continuum in terms of probabilities of respondents endorsing response formats with higher scores), monotonicity (whether the probability of obtaining high scores on an item does not decrease as latent trait scores increase), and local independence (whether associations between items are explained only by their relationship with the construct). If these conditions are met, the items fit the monotone homogeneity model (MHM), and can thus be considered a scale. In the case of polytomous items (as for I-eHEALS), if they also meet a further condition, invariant item ordering (IIO) (ie, items show the same ordering of difficulty across different levels of the latent), the scale allows the identification of “person-free” hierarchy of item difficulty. Therefore, the scale can be used for comparing subgroups regarding their position on the latent trait [36]. Unidimensionality was tested by examining homogeneity—indicating the degree of association between all items (*H*), and between each item and the item set (*H*_i_)—and by performing an automated item selection procedure (AISP), which is the bottom-up item clustering algorithm performed for increasing homogeneity thresholds [37]. Recommended thresholds for homogeneity (range 0-1) are 0.3 to 0.4 (weak), 0.4 to 0.5 (medium), and over 0.5 (good). Local independence, monotonicity, and IIO were tested via check.ca, check.monotonicity, and check.iio functions; output was examined for significant violations of these assumptions [24,35]. The minimum size of the restscore group (*minsize*) was set at 30 because of the small size of our sample [24]. Person fit was assessed by computing the number of Guttman errors per participant [38].

Additionally, we performed Rasch analyses in line with prior explorations by Nguyen and colleagues [23] and guidelines by Tennant and Conaghan [26] using the Winsteps software. Fit to the RSM was examined, as the 8 items use the same response scale and RSM is a more parsimonious model for this format [39]. We examined item and person infit and outfit against an acceptable mean squares range of 0.6-1.4 and standardized fit statistics of +/−2.0 (Wright and Linacre [40]). Two criteria for good item rating structure were examined: 10 or more observations in each rating category; and outfit mean-squares <2.0 for each category. The hierarchy of item difficulty and the match between person ability and item difficulty (scale targeting) were explored graphically. Person reliability (adequate values >.85) and person separation (>2.5) were computed. Differential item functioning (DIF) was examined for differences in item difficulty between groups against a threshold of >0.5 logits for gender, age (dichotomized using median split), education level (college versus no college education), and source of data (Study 1 or 2).

#### Classical Test Theory Analyses

Reliability of the final scale was assessed using Cronbach alpha [41]. Bivariate correlations and independent samples *t* test were used to assess differences in mean I-eHEALS scores related to gender, age, educational levels, and frequency of Internet use. Convergent validity of the scale was assessed by computing Pearson correlations between I-eHEALS and other constructs which have been shown to be positively correlated with eHealth literacy in past research, such as attitudes toward eHealth or perceived outcomes of Web-based health information seeking.

## Results

### Items Characteristics

Participants scored on average 26.65 (SD 6.276) on I-eHEALS. No differences were found among Study 1 (mean 27.21, SD 6.083) and Study 2 (mean 26.27, SD 6.388) participants; *t*_294_=1.261, *P*=.21. Average scores on the individual I-eHEALS items ranged between 2.75 (SD 1.146, item 8) and 3.62 (SD 0.960, item 2) on a 1 to 5 scale, thus indicating considerable variation in item difficulty. Interitem correlations ranged from *r*=.309 (*P*<.001) to *r*=.800 (*P*<.001). All items except one (*Strongly Disagree* category for item 2, 8 observations) had at least 10 observations for each category. More details on items characteristics and inter-item correlations are presented in Table 1.

**Table 1:** Descriptive statistics and inter-item correlations for the Italian version of the eHealth Literacy Scale (I-eHEALS) items.

Item	Mean (SD^a^)	Skew	Kurt	Inter-item correlations							
				1	2	3	4	5	6	7	8
1	3.56 (0.996)	−0.59	−0.10	1							
2	3.62 (0.960)	−0.62	0.06	.800	1						
3	3.23 (0.969)	−0.12	−0.45	.601	.614	1					
4	3.40 (0.982)	−0.45	−0.29	.717	.692	.661	1				
5	3.52 (0.978)	−0.60	0.11	.578	.603	.519	.579	1			
6	3.16 (1.171)	−0.28	−0.84	.386	.378	.353	.438	.468	1		
7	3.41 (1.107)	−0.43	−0.49	.356	.372	.309	.406	.407	.719	1	
8	2.75 (1.146)	0.12	−0.88	.445	.461	.450	.488	.513	.529	.530	1

^a^SD: standard deviation.

### Exploratory and Confirmatory Factor Analyses

All the items correlated at least .3 with at least one other item, suggesting reasonable factorability (Table 1). The Kaiser-Meyer-Olkin measure of sampling adequacy was .879, above the commonly recommended value of .6, and Bartlett test of sphericity was significant (χ^2^_28_=1368.7, *P*<.001). FA was thus deemed to be suitable with all 8 items. A total of 13 multivariate outliers were identified (Mahalanobis distance >26.125 chi-square threshold for df=8, *P*=.01); these were kept in the dataset to replicate procedures of published factor analyses.

PCA suggested a 2-factor solution with a first factor explaining 57.7% of the variance in I-eHEALS scores, and a second factor explaining an additional 14.9% of the variance (see Table 2 for details). All items presented high factor loadings on Factor 1 (range=.651 to .834), whereas two items presented high factor loadings also on Factor 2 (item 6=.585, item 7=.626). This 2-factor solution mirrors the one proposed by Soellner and colleagues [14].

**Table 2:** Principal components analysis of the Italian version of the eHealth Literacy Scale (I-eHEALS) items. Factor loadings <.4 are not displayed.

Item	Factor 1	Factor 2
I-eHEALS1	.821	
I-eHEALS2	.827	
I-eHEALS3	.751	
I-eHEALS4	.834	
I-eHEALS5	.773	
I-eHEALS6	.683	.585
I-eHEALS7	.651	.626
I-eHEALS8	.717	
Eigenvalue	4.619	1.196
Cumulative explained variance	57.7%	72.7%

CFA was run for two different models: the 1-factor model proposed by Norman and Skinner (Model I) and the 2-factor model proposed by Soellner et al and suggested by our own PCA (Model II). The comparison of the two models showed better fit for the 2-factorial model (Table 3). This was confirmed by the individual model indices and by chi-square differences of 144.8 (df=1; *P*<.001) for Model I versus Model II.

Although Model II appeared to be the one better fitting our data, chi-square tests and fit indices (with the exception of SRMR) indicated a suboptimal model fit for both models.

**Table 3:** Confirmatory factor analysis of two models of the Italian version of the eHealth Literacy Scale (I-eHEALS).

Model	Chi-square (df)^a^	CFI^b^	TLI^c^	RMSEA^d^	SRMR^e^	AIC^f^	BIC^g^
Model I 1 factor (Norman and Skinner)	247.8 (20)	0.833	0.766	0.196	0.098	5767.609	5826.655
Model II 2 factors (Soellner et al and PCA^h^)	102.9 (19)	0.938	0.909	0.122	0.069	5624.841	5687.577

^a^Chi-square difference: Model I versus Model II, χ^2^_1_=144.77; *P*<.001, N=296.

^b^CFI: comparative fit index.

^c^TLI: Tucker-Lewis index.

^d^RMSEA: root mean squared error of approximation.

^e^SRMR: standardized root mean squared residual.

^f^AIC: Akaike information criterion.

^g^BIC: Bayesian information criterion.

^h^PCA: principal component analysis.

### Nonparametric Item Response Theory: Unidimensionality, Local Independence, Monotonicity, and Invariant Item Ordering

The *H*_i_ values of all the items of I-eHEALS and the summary *H* coefficient of the scale (*H*=0.553, SE 0.032) were above the lower cutoff point of 0.3 (see Table 4). These results confirmed that the I-eHEALS scale can be considered unidimensional, and that all the items measure a single underlying construct as intended.

Exploration of the scale unidimensionality with increasing homogeneity thresholds via *AISP* indicated that at homogeneity threshold levels of 0.30 to 0.45, all items belonged to the same scale; whereas at a threshold of 0.50, items 6 and 7 clustered together in a separate scale.

**Figure 1:**
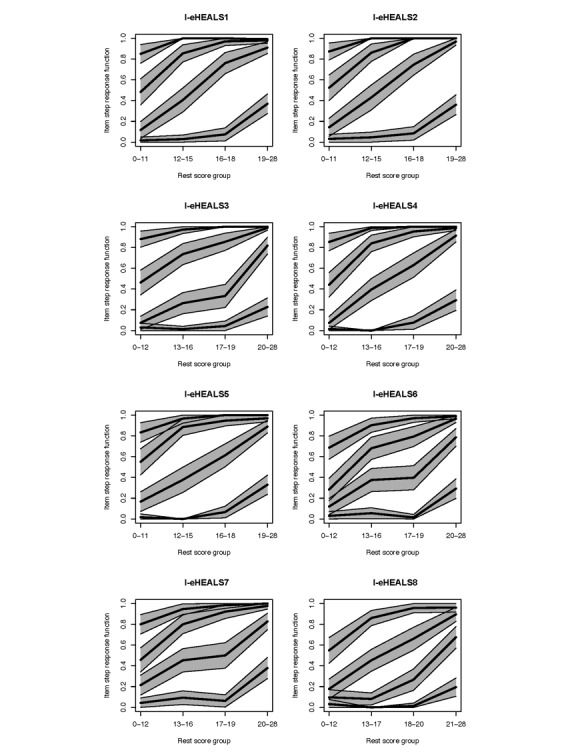
Italian version of the eHealth Literacy Scale (I-eHEALS) item step response functions.

Local independence and monotonicity tests suggested no significant violations of these two criteria for any of the items in the dataset, thus confirming that no conditional associations are present between the items except those due to the latent dimension, and that the probability of endorsing response options indicating higher ability increases monotonically for all items as respondents’ level of eHealth literacy increases (Figure 1).

**Table 4:** Loevinger’s scalability coefficients for Italian version of the eHealth Literacy Scale (I-eHEALS) items.

Item	H_i_^a^	SE^b^
I-eHEALS1	0.585	0.036
I-eHEALS2	0.599	0.033
I-eHEALS3	0.546	0.044
I-eHEALS4	0.604	0.034
I-eHEALS5	0.560	0.039
I-eHEALS6	0.516	0.043
I-eHEALS7	0.486	0.042
I-eHEALS8	0.541	0.040
Scale	H^c^	SE
I-eHEALS scale	0.553	0.541

^a^H_i_: item homogeneity.

^b^SE: standard error.

^c^H: scale homogeneity.

The results of IIO assessment indicated no significant violations of this assumption. Thus, the items showed the same order of difficulty across levels of the latent construct, with item 2 as easiest and item 8 as most difficult.

A total of 18 participants had a number of Guttman errors at the extreme high end of the distribution (higher than 1.5 interquartile ranges above the third quartile), and were considered outliers.

### Parametric Item Response Theory: Item Properties Within the Rasch Framework

The item infit and outfit and standardized *t* scores are shown in Table 5. All item fit mean squares were within the accepted range. Underfit (both mean squares >1.4 and standardized fit statistics >2) was identified in 28 persons (9.5%), and 45 (15.2%) overfitted the model (both mean squares <.60 and standardized fit statistics <−2) according to infit values. These results were largely consistent with results for outfit values.

All categories had 10 or more observations, except the SD category for item 2 (8 observations). Outfit mean-squares for each rating category were within the accepted range.

**Table 5:** Italian version of the eHealth Literacy Scale (I-eHEALS) item infit and outfit and standardized *t* scores.

Item	Measure	Model SE^a^	Infit MSQ^b^	ZSTD^c^	Outfit MSQ	ZSTD
I-eHEALS1	−0.49	0.09	0.8411	−1.9392	0.8458	−1.8092
I-eHEALS2	−0.62	0.09	0.782	−2.7092	0.7468	−3.0693
I-eHEALS3	0.22	0.08	0.9237	−0.9291	0.9461	−0.6291
I-eHEALS4	−0.12	0.09	0.7068	−3.8793	0.698	−3.9193
I-eHEALS5	−0.4	0.09	0.8903	−1.3091	0.8549	−1.7091
I-eHEALS6	0.38	0.08	1.3131	3.5213	1.3256	3.5913
I-eHEALS7	−0.15	0.09	1.37	3.9814	1.3018	3.2313
I-eHEALS8	1.18	0.08	1.1172	1.4411	1.1209	1.4511

^a^SE: standard error.

^b^MSQ: mean-square.

^c^ZSTD: z-standardized.

**Figure 2:**
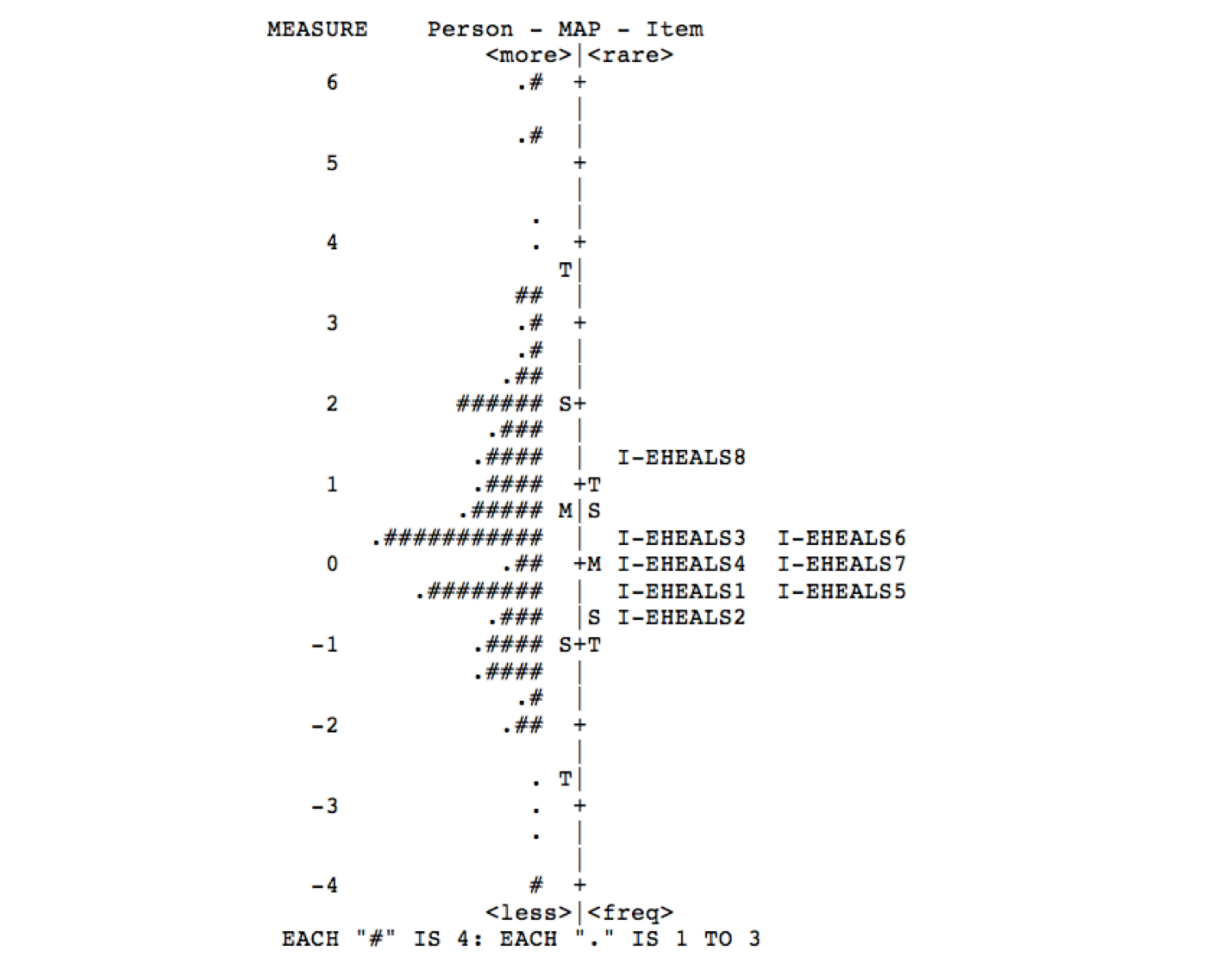
Hierarchy of Italian version of the eHealth Literacy Scale (I-eHEALS) item difficulty and targeting of items and persons.

The hierarchy of item difficulty (from the easiest to most difficult—item 2 to item 8) and targeting of items and persons are shown in Figure 2. Most participants were located at above average levels of the eHealth literacy latent, whereas items and item category thresholds were located predominantly close to average values. Thus, I-eHEALS was less able to measure respondents with extreme levels of eHealth literacy. In total, 5 maximum scores and 3 minimum scores were identified, indicating limited ceiling and floor effects.

The real person reliability was .87 (person separation 2.57), indicating good ability to distinguish between respondents of different ability levels.

DIF was identified for item 8 by gender (difficulty higher by 0.59 logits for women; *P*<.001), and for item 7 depending on the source of data (difficulty higher by 0.56 logits in Study 1 than in Study 2; *P*=.002). No differences in item difficulty were present between respondents aged below 33 years or above 33 years, and between participants with college versus no college education.

### Classical Test Theory: Reliability and Validity

The final I-eHEALS (mean 26.64, SD 6.276) had excellent reliability (Cronbach alpha=.891). Pearson correlations and *t* test to assess differences in I-eHEALS scores related to gender, age, educational level, and frequency of Internet use were performed. None of the respondents’ characteristics under investigation was found to be significantly associated with I-eHEALS scores (Table 6). Pearson correlations between respondents’ I-eHEALS scores and scores on other theoretically correlated constructs showed positive and significant correlations with health information seeking on the Web (*r*=.434, *P*<.001), trust in the Internet as a source of health information (*r*=.251, *P*=.006), attitudes toward the adoption of the ICTs for health purposes (*r*=.479, *P*<.001), eHealth predisposition (*r*=.377, *P*<.001), use of Internet searching strategies (*r*=.453, *P*<.001), perceived outcomes of seeking health information by surfing the net (*r*=.577, *P*<.001), and use of Internet evaluation criteria (*r*=.331, *P*<.001).

**Table 6:** Association of Italian version of the eHealth Literacy Scale (I-eHEALS) scores with theoretically relevant variables (N=117).

Characteristics	I-eHEALS	
	*d* ^a^	*P*
Gender	.202	.28
	*r* ^b^	*P*
Age	.076	.42
Educational level	.066	.48
Internet use	.051	.58
Web-based health information seeking	.434	<.001
Trust in the Internet as a source of health information	.251	.006
Attitudes toward the adoption of ICTs^c^for health purposes	.479	<.001
Perceived outcomes	.577	<.001
eHealth predisposition	.377	<.001
Use of searching strategies	.453	<.001
Use of evaluation criteria	.331	<.001

^a^*d*: Cohen *d* effect size.

^b^*r*: Pearson correlation coefficient.

^c^ICT: information and communication technology.

## Discussion

### Principal Findings

The main aim of this study was to validate I-eHEALS. As previous validation studies did not agree on the factor structure of the scale, particular attention was devoted to the investigation of this aspect. In addition to CTT and factor analyses, IRT techniques were therefore applied to take into account the fact that the items of the scale might differ in terms of difficulty, as it has been recommended for constructs measuring abilities [42]. I-eHEALS scale scores were used to examine group differences and associations with theoretically related concepts.

Although health literacy tools have been examined with other statistical techniques [43,44], most measure developments and validations in the field still rely on less adequate FA methods [45]. Even though some recent studies have already applied IRT to the original eHEALS [23,46], our study was the first to apply these techniques to investigate the structure of a translation of the scale. Also, in contrast with other validations which were conducted in students or in patients’ samples, we used a general population sample. Following the results of preliminary exploratory and confirmatory factor analyses, the scale appeared to have a 2-factor structure. This solution had already been proposed in the past by Soellner and colleagues [14], and the existence of a second dimension was also reported for the Dutch eHEALS version [11]. Borrowing terminology widely used in conceptualizations of traditional health literacy [47], the two dimensions suggested by PCA seemed to refer to *functional eHealth literacy* skills (items 1-5 and 8) and *critical eHealth literacy* skills (items 6 and 7), respectively. However, when CFA was performed to compare the fit of model resulting from our PCA and proposed by Soellner and colleagues [14] and of the single-factor model originally proposed by the authors of the scale [7], neither model showed an adequate fit to the data.

The dimensionality of the scale was therefore subsequently assessed using nonparametric and parametric IRT methods, which take into account the fact that the items might differ in terms of difficulty. Such an approach has been recommended for constructs measuring abilities like eHEALS [42]. Mokken analyses showed that the Italian eHEALS version can be considered unidimensional, and that all the items measure a single underlying construct with good homogeneity, in line with what was originally proposed by the authors of the scale [7]. Moreover, our analyses showed that the data fit the MHM and also meet the additional assumption of IIO. There are three key implications of this result for the applicability of the scale (see [24] for a theoretical overview). First, I-eHEALS items can be used to order respondents with respect to their latent eHealth literacy levels based on the scale score, thus justifying the use of mean scores for further analyses. Simply examining reliability via Cronbach alpha is not by itself sufficient to allow the use of mean or sum scores [48], and a confirmation of unidimensionality and monotonicity is necessary before considering its use as an indicator of reliability [49]. Second, fitting the MHM model implies that the test is able to order respondents on the latent measurement continuum in a similar way if different subsets of items are used (and thus achieving item-free measurement). This suggests that the items are a good starting point for developing a larger item pool, from which alternative questionnaire versions can be developed, for instance, for repeated assessments in longitudinal studies. Third, IIO implies that the items target eHealth literacy skills that form the same hierarchy for all respondents (ie, ordering holds at individual level as well). This allows the use of I-eHEALS to compare subgroups of citizens with different levels of eHealth literacy, which is a common aim of health literacy research [50].

The stricter Rasch analyses additionally allowed us to conclude that I-eHEALS has a good ability to distinguish between respondents of different ability levels and that only a few differences in item difficulty were present between male and female respondents, whereas no such differences were found for younger versus older participants or between participants with college versus no college education. Rasch analyses also indicated that the scale is less able to measure respondents with extreme levels of eHealth literacy. Interestingly, the order of difficulty of our items was different from that identified in other studies using IRT on eHEALS [23,46], thus suggesting that some personal characteristics might play a role in the kind of eHealth literacy tasks people perceive as more or less demanding. Participants in our sample rated item 2 as the easiest and item 8 as the most difficult. The Health Science college students in the study by Nguyen et al [23] perceived item 4 to be the easiest item and item 5 to be the hardest item. Within the same study, participants recruited from Amazon MTurk rated item 7 as the easiest item and item 6 as the most difficult one. As acknowledged by Nguyen et al, these differences could be attributed to the demographic makeup of each sample group: Health Science students may be more familiar with the location of health resources on the Internet, whereas tech-savvy and highly educated MTurkers might have higher perceptions of their ability to distinguish high-quality health information versus low-quality health information. We used a general population sample (participants were neither health nor technology experts) and it is therefore reasonable that our participants perceived different tasks as being more or less demanding. We strongly encourage future research to investigate these aspects in more depth.

Consistently with other translated versions of eHEALS, no significant correlations of I-eHEALS scores with respondents’ characteristics were found. Although a rigorous test of this relationship (or lack thereof) would require a more diverse sample as regards age and education, the absence of an association with the traditional determinants of health literacy might be seen as a further indication that eHEALS (and its translations) is not able to capture actual skills. This hypothesis would be supported by a study conducted in two Dutch populations by van der Vaart and colleagues [11]. The authors found no association between scores on eHEALS and an actual Internet performance test, questioning the ability of the instrument to adequately capture the phenomenon under investigation. In this view—as it has been argued in the past—eHEALS could be more realistically described as a measure of self-efficacy in the electronic health information context [11,51]. As suggested by Frisch and colleagues [52], this is a common shortcoming of self-reported measures of health literacy. In our view, however, this does not undermine the value of eHEALS. According to one of the eHEALS authors, the nonsignificant correlation could be related to the fact that the scale in its present form does not capture the skills related to the use of social media, which have become more and more important in the last few years. In this perspective, eHEALS can still be considered a valid tool for assessing competency with Web 1.0 technologies [53]. Higher I-eHEALS scores were indeed shown to be significantly associated with more frequent Web-based health information seeking, higher trust in the Internet as a source of health information, more positive attitudes toward the adoption of ICTs for health purposes, higher eHealth predisposition, and more positive perceived outcomes of seeking health information by surfing the net. These associations suggest that the scale can safely be used to assess consumers’ perceived comfort and skills in using information technology for health. This would be useful to identify those who may be keener to participate in eHealth interventions or use eHealth resources within a clinical environment and those who are in need of more support.

### Limitations

Three limitations of this study have to be acknowledged. First, our sample was younger, more educated, and included a higher percentage of women compared with the general population of the Italian-speaking region of Switzerland [54]. If we consider, however, that these are the characteristics that are usually associated with health information seeking on the Web (eg, [4]), we believe that our sample is suitable to provide us with an adequate snapshot of our population of interest. Yet, being a convenience sample, it cannot be considered truly representative of the population, thus limiting the generalizability of our results. Second, our sample was relatively small compared with other validations of translated version of eHEALS. However, NIRT can cope with small sample sizes better than other statistical techniques [21]. We are therefore confident that sample size had a limited impact on our results, particularly given the small number of items investigated. Finally, we did not include a measure of actual ability to perform eHealth literacy tasks. However, as our goal was to provide a good set of items for investigating eHealth literacy in Italian-speaking populations, this was outside the scope of this study. Nevertheless, we join other scholars in acknowledging the need for more research specifically aimed at further investigating the link between actual and perceived ability to perform eHealth literacy tasks—as it was done by van der Vaart and colleagues [11] in the Netherlands. Only after doing that, it will be possible to fully capture the complexity of the phenomenon under investigation.

### Conclusions and Practice Implications

The study confirmed that I-eHEALS is a reliable and valid tool to assess Italian-speaking consumers’ perceived comfort and skills in using information technology for health. A previous validation of I-eHEALS among students had already proven the suitability of the scale among that specific population [16]. The sample used in our study allows us to extend this conclusion to the general population. I-eHEALS can therefore safely be used by public health officials and health care providers to identify those who are most ready to take part in eHealth interventions or to use Web-based resources within both clinical and nonclinical environments, as well as those who would need more support. Moreover, compared with the previous Italian validation, our IRT analysis was also able to highlight several strengths of the scale, for instance, its unidimensionality which justifies the calculation of a total mean score of all the items. Moreover, it indicated future directions in eHealth literacy assessment, such as the importance of wording in item development, and the possibility of extending the item pool and developing alternative versions.

## Appendix

Multimedia Appendix 1 The Italian version of the eHealth Literacy Scale (I-eHEALS).

Multimedia Appendix 2 Overview of the scales used in the study.

## Abbreviations

AIC: Akaike information criterion
AISP: automatic item selection procedure
BIC: Bayesian information criterion
CFI: comparative fit index
CFA: confirmatory factor analysis
CTT: classical test theory
DIF: differential item functioning
DMM: Double Monotonicity Model
EFA: exploratory factor analysis
eHEALS: eHealth Literacy Scale
FA: factor analysis
HRI: Human Research Act
ICT: information and communication technology
I-eHEALS: Italian version of the eHealth Literacy Scale
IIO: invariant item ordering
IRT: item response theory
MHM: monotone homogeneity model
MSA: Mokken scale analysis
NIRT: nonparametric item response theory
PCA: principal component analysis
RMSEA: root mean squared error of approximation
RSM: rating scale model
SRMR: standardized root mean squared residual
TLI: Tucker-Lewis index
