# Supplementary material for: A Psychometric Analysis of the Italian Version of the eHealth Literacy Scale Using Item Response and Classical Test Theory Methods
Source: J Med Internet Res. 2017 Apr 11;19(4):e114. doi: 10.2196/jmir.6749 (PMC5405289; doi:10.2196/jmir.6749)
Supplement: Multimedia Appendix 1 [file jmir_v19i4e114_app1.pdf]

Multimedia Appendix 1. The Italian version of the eHealth Literacy Scale (I-eHEALS).

Item	Italian translation[a]	Original version[b]
I-eHEALS1	So come trovare informazioni utili riguardanti la salute in Internet	I know how to find helpful health resources on the Internet
I-eHEALS2	So come utilizzare Internet per trovare risposte alle mie domande sulla salute	I know how to use the Internet to answer my health questions
I-eHEALS3	So quali informazioni riguardanti la salute sono disponibili in Internet	I know what health resources are available on the Internet
I-eHEALS4	So dove trovare informazioni utili riguardanti la salute in Internet	I know where to find helpful health resources on the Internet
I-eHEALS5	So come utilizzare le informazioni riguardanti la salute trovate in Internet per aiutarmi	I know how to use the health information I find on the Internet to help me
I-eHEALS6	Ho le competenze necessarie per valutare le informazioni riguardanti la salute che trovo in Internet	I have the skills I need to evaluate the health resources I find on the Internet
I-eHEALS7	Sono in grado di distinguere informazioni riguardanti la salute di alta qualità da quelle di bassa qualità presenti in Internet	I can tell high quality from low quality health resources on the Internet
I-eHEALS8	Mi sento sicuro/a nell'utilizzare informazioni ottenute da Internet per prendere decisioni riguardanti la salute	I feel confident in using information from the Internet to make health decisions

Notes: [a] As in the English version, answers are on a 5-point Likert scale ranging from 1 = *In completo disaccordo* [Strongly disagree] to 5 = *Completamente d'accordo* [Strongly agree]; [b] Source: Norman, C. D., & Skinner, H. A. (2006a). eHEALS: The eHealth literacy scale. *Journal of Medical Internet Research, 8*(4), e27.
