# Supplementary material for: A Psychometric Analysis of the Italian Version of the eHealth Literacy Scale Using Item Response and Classical Test Theory Methods
Source: J Med Internet Res. 2017 Apr 11;19(4):e114. doi: 10.2196/jmir.6749 (PMC5405289; doi:10.2196/jmir.6749)
Supplement: Multimedia Appendix 2 [file jmir_v19i4e114_app2.pdf]

Multimedia Appendix 2. Overview of the scales used in the study

Construct	Items	Response options	Source
Online health information seeking	- How often do you get health information from the Internet’	5-point scale (Less than once a month – Every day)	-
Trust in the Internet as a source of health information	- How much do you trust health information from the Internet?	5-point scale (Not at all – Very much)	-
Attitudes towards the adoption of ICTs for health purposes	- I am in favor of using the Internet for health-related purposes<br>- I am in favor of using social media (e.g., Facebook or Twitter) for health-related purposes	7-point scale (Strongly disagree – Strongly agree)	-
Use of Internet searching strategies	In order to find health information on the Internet you usually do the following:<br>- use a site that my physician recommended<br>- follow links that appear on websites<br>- ask questions in forums<br>- use my Favorites list<br>- use a site that a friend recommended	5-point scale (Never – Always)	[10]
Perceived outcomes of seeking health information by surfing the net	Do you agree or disagree that seeking health information on the Internet…<br>- improved your ability to manage your health needs<br>- enabled you to ask your physician questions resulting from the information you acquired on the Internet<br>- enabled you to show your physician the information that you retrieved<br>- raised your sense of power in your encounter with the physician<br>- improved your understanding of the symptoms, conditions, or treatments in which you were interested<br>- updated your knowledge in health innovations<br>- led you to take independent steps (such as seeing a specialist, or	5-point scale (Strongly disagree – Strongly agree)	[10, 28]

	changing an exercise regimen or eating habits)<br>- enabled you to think about alternative treatment options; and made you more aware of patients' insurance rights		
Use of Internet evaluation criteria	How important are the 5 following criteria in judging a website:<br>- the purpose of the site is clearly stated and the information is accurate<br>- it has a reliable source; a contact is available for questions/comments/help<br>- retrieval is easy and can be done in a timely manner<br>- the scope of information suits my needs	5-point scale (Not at all important – Very important)	[29]
Predisposition towards eHealth	- How useful do you feel the Internet is in helping you in making decisions about your health?<br>- How important is it for you to be able to access health resources on the Internet?	5-point scale (Not at all useful/important – Very useful/important)	[7]
